# Genome sequence of dengue virus serotype 1 isolated from a pediatric patient enrolled in an Ivermectin trial in Thailand

**DOI:** 10.1128/mra.00523-24

**Published:** 2024-09-24

**Authors:** Preeyanuch Sayboonruan, Adisak Songjaeng, Kwanrutai Chin-Inmanu, Phitchapha Proykhunthod, Dararat Prayongkul, Ranyikar Poraha, Dumrong Mairiang, Keswadee Lapphra, Kulkanya Chokephaibulkit, Nuntaya Punyadee, Panisadee Avirutnan

**Affiliations:** 1 Division of Dengue Hemorrhagic Fever Research, Research Department, Faculty of Medicine Siriraj Hospital, Mahidol University, Bangkok, Thailand; 2 Siriraj Center of Research Excellence in Dengue and Emerging Pathogens, Faculty of Medicine Siriraj Hospital, Mahidol University, Bangkok, Thailand; 3 Division of Medical Bioinformatics, Research Group and Research Network Division, Research Department, Faculty of Medicine Siriraj Hospital, Mahidol University, Bangkok, Thailand; 4 Molecular Biology of Dengue and Flaviviruses Research Team, Medical Molecular Biotechnology Research Group, National Center for Genetic Engineering and Biotechnology, National Science and Technology Development Agency, Bangkok, Thailand; 5 Department of Pediatrics, Faculty of Medicine Siriraj Hospital, Mahidol University, Bangkok, Thailand; 6 Siriraj Institute of Clinical Research (SICRES), Faculty of Medicine Siriraj Hospital, Mahidol University, Bangkok, Thailand; Katholieke Universiteit Leuven, Leuven, Belgium

**Keywords:** dengue virus genome, pediatric patient, clinical trial, dengue fever

## Abstract

We sequenced the genome of dengue virus serotype 1 (DENV1) strain 4101301, isolated from a child with dengue fever in Thailand and cultured in C6/36 mosquito cells. These data are crucial for studying DENV1’s genetic diversity, evolution, and epidemiology and advancing the knowledge for developing antiviral drugs and vaccines targeting DENV.

## ANNOUNCEMENT

Dengue virus (DENV) significantly impacts global health, causing over 390 million infections each year. The infection may result in dengue fever (DF), dengue hemorrhagic fever, and dengue shock syndrome (1). DENV comprises four serotypes and belongs to the *Flaviviridae* family and *Flavivirus* genus. Its genome consists approximately of 10.7 kilobases of positive single-stranded RNA. Dengue vaccines and treatments are difficult to develop partly due to the complex interaction of viral strains and immune responses. Thus, the genetic diversity of dengue virus strains still requires continuing investigation.

The DENV1 strain 4101301 was isolated from a plasma sample collected on the first day of enrollment from an 8.2-year-old female patient with mild DF in the PKIDEN clinical trial (ClinicalTrials.gov ID: NCT03432442) in 2018. This patient presented with a secondary DENV infection. The virus was isolated using standard protocols with C3/36 mosquito cell cultures (2) and passaged three times in C6/36 cells. Viral RNA was extracted from the culture supernatant 6 days post-inoculation using the MagLead 12gC system and MagDEA Dx SV Kit (Precision System Sciences, Japan). Then, RNA was reverse transcribed into cDNA using Random Hexamer and the ProtoScript II First Stand cDNA Synthesis Kit (New England Biolabs, USA). The reverse transcription was performed at 42°C for 90 minutes. Then, cDNA was subjected to PCR amplification. Eleven overlapping DENV1-specific primers (https://github.com/si-medbif/virus_seq/tree/main/Dengue_Virus/DENV_multiplex_tiling_PCR/), designed with PrimalScheme (3), were used with Q5 High-Fidelity DNA polymerase (New England Biolabs, USA) to generate ~1.2 kb amplicons. PCR conditions included an initial denaturation at 98°C for 30 seconds, followed by 40 cycles of 98°C for 15 seconds and 65°C for 5 minutes. PCR products were quality assessed via agarose electrophoresis before pooling for sequencing. Sequencing was performed using the Oxford Nanopore Technology platform with the SQK-RBK114-24 kit with R10.4.1 flow cell, following the rapid barcoding protocol. The sequencing data were analyzed according to the Artic protocol version 1.2.3 (4). Basecalling was performed with MinKNOW version 5.5.5 using the super-accurate model. Primer and adaptor sequences were removed with Guppy version 6.5.7, and reads were filtered to 400–1,700 bp using ARTIC guppyplex. The output included 1,530 reads, 1,274,580 bases, and an *N*_50_ of 964 bp. A 10,724-bp consensus sequence was generated using Medaka version 1.11.3 (5), covering the coding region, 5′ UTR, and nearly complete 3′ UTR, with 46.7% GC content. All reads were mapped to the consensus sequence using Minimap2 (6), giving an average depth of 101×. Pairwise alignment with BLASTn (7) showed 99% identity to the 2016 DENV1 isolate 48555 from Singapore (accession no. MF033260). The translated protein sequence [Expasy Translate tool (8)] was compared to our laboratory DENV1 Hawaii strain (polyprotein accession no. QBK46949) using BLASTp (9). Notable amino acid differences were found in the capsid, envelope, and NS1 proteins (Table 1). These findings underscore the importance of characterizing circulating DENV strains, particularly for genes involved in viral replication and immunopathogenesis, as this information is crucial for developing vaccines and therapies.

**TABLE 1:** Amino acid differences between DENV1 Hawaii strain (laboratory reference) and DENV1 clinical strain 4101301

Gene	Gene length (amino acid)	Amino acid identity (%)	Number of amino acid difference	Percentage of amino acid divergence
Capsid (C)	114	95.61	5	4.38
prM	166	97.59	4	2.41
Envelope (E)	495	96.97	15	3.03
NS1	352	96.87	11	3.12
NS2A	218	98.62	3	1.37
NS2B	130	99.23	1	0.76
NS3	619	98.38	10	1.61
NS4A	127	97.63	3	2.36
protein_2k	23	100.00	0	0.00
NS4B	249	99.19	2	0.80
NS5	899	98.22	16	1.78

## Data Availability

The genome sequence of DENV1 strain 4101301 is available in the NCBI GenBank under the accession number PP773768. The raw sequencing data have been deposited in the Sequence Read Archive (SRA) database under the BioProject accession number PRJNA1118487 (SRA accession number SRR29243816).
